# Construction and validation of a diagnostic model for high-risk papillary thyroid microcarcinoma

**DOI:** 10.3389/fendo.2024.1431584

**Published:** 2024-10-09

**Authors:** Yu Liang, ErXi Fan, Jing Zhang, Tong Xu, Jun Song, Fuhong Huang, Dong Wang

**Affiliations:** Department of Ultrasound, Sichuan Provincial People's Hospital, School of Medicine, University of Electronic Science and Technology of China, Chengdu, Sichuan, China

**Keywords:** papillary thyroid microcarcinoma, high risk, diagnostic model, ultrasound, clinical application

## Abstract

**Objective:**

The purpose of this study was to construct a diagnostic model by exploring the potential predictors of high-risk Papillary Thyroid Microcarcinoma (PTMC) and verifying its reliability.

**Methods:**

A retrospective analysis of PTMC patients who underwent surgical treatment from 2004 to 2015 in the SEER database (training set) and the clinical pathological ultrasound information of PTMC patients at the Sichuan Provincial People's Hospital from 2020 to 2022 (external validation set) was conducted. In the training set, univariate and multivariate logistic regression analyses were used to screen independent predictive factors for high-risk PTMC patients in pathology. A nomogram diagnostic model was further constructed. Additionally, ROC curves and calibration curves were drawn to evaluate the efficiency of the model. In the external validation set, the diagnostic model was indirectly evaluated based on preoperative ultrasound imaging features to explore the feasibility and reliability of diagnosing high-risk PTMC through preoperative ultrasound imaging features.

**Results:**

A total of 1628 patients were included in the training set, and 530 patients were included in the test set. The independent risk factors for pathological high-risk PTMC were sex, age, tumor maximum diameter, tumor invasive, and cervical lymph nodes (*P*<0.05). The C-index of the nomogram constructed based on these five factors was 0.947, with an optimal sensitivity of 96.7% and a specificity of 86.0%. The calibration curve showed that the model had high consistency. The area under the curve (AUC) value of the ROC curve for high-risk PTMC predicted by the risk score based on ultrasound features was 0.824 [95% CI (0.789, 0.860)], which was highly consistent with the risk score based on pathological features (κ= 0.758, *P*<0.05).

**Conclusion:**

Indirect evaluation of a high-risk PTMC diagnostic model based on preoperative ultrasound imaging features had high predictive efficiency and potential value for clinical application.

## Introduction

Thyroid carcinoma, especially Papillary Thyroid Microcarcinoma (PTMC), is suspected to be overdiagnosed and overtreated worldwide (1). For low-risk PTMC, active monitoring therapy is recommended in clinical practice, and surgical treatment is not necessary (2). However, currently, only postoperative pathology can distinguish low-risk PTMC from high-risk PTMC (3). There are no imaging standards for diagnosing low-risk PTMC and high-risk PTMC before surgery (4). This study established a pathological diagnostic model based on the SEER database to diagnose high-risk PTMC and indirectly evaluated the model through the characteristics of thyroid and cervical lymph node ultrasound imaging, which aims to provide a diagnostic reference for clinicians to determine individualized treatment strategies.

## Materials and methods

### SEER database case data acquisition

We used SEER*Stat 8.4.1 software to extract, download and screen data from the Incision -Seer Research Data, 17 Registries, Nov 2022 Sub (2000-2020). The clinical pathological characteristics of thyroid cancer patients with surgical treatment, including age, sex, race, surgical method, pathological characteristics, and prognosis, were selected. Inclusion criteria: ① The pathological type is papillary thyroid carcinoma (PTC), ICD-O-3 codes included 8050 (papillary carcinoma), 8260 (papillary adenocarcinoma), 8340 (papillary carcinoma follicular subtype), 8341 (micropapillary carcinoma), 8342 (papillary carcinoma eosinophil subtype), 8343 (papillary carcinoma enveloping subtype), and 8344 (papillary carcinoma columnar cell subtype); ② The diagnosis was made between 2004 and 2015; ③ Race was Asian or Pacific Island; ④ Surgical treatment of PTC and cervical lymph node biopsy; ⑤ Pathological results: The maximum diameter of the tumor was ≤ 10 mm; ⑥ The tumor capsule invasion was clearly recorded; ⑦ cervical lymph node metastasis was clearly recorded; ⑧ The number of tumors were clearly recorded. Exclusion criteria: ① Failure to undergo surgical treatment for PTC; ② failure to perform cervical lymph node dissection and pathological assessment of cervical lymph node status; ③ other types of thyroid malignancies (follicular thyroid cancer, medullary thyroid cancer, undifferentiated thyroid cancer, etc.) or without histological records; ④ specific values for tumor maximum diameter >10 mm or no tumor size; ⑤ lack of complete pathological feature description records; ⑥ unknown survival status.

### Clinical materials

We conducted a retrospective analysis of the clinical pathological ultrasound data of PTMC patients who underwent surgical treatment at the Thyroid Diagnosis and Treatment Center of Sichuan Provincial People's Hospital from January 2020 to December 2022. The inclusion criteria were as follows: ① Preoperative ultrasound imaging examination of thyroid nodules and cervical lymph nodes, confirmation of the ultrasound diagnostic conclusion, and high-quality retained ultrasound images. ② The postoperative pathological description of the thyroid nodules and cervical lymph nodes was accurate, and the pathological diagnosis was clear. ③ The postoperative maximum diameter of the thyroid nodules was ≤10 mm.④ Clear localization of the nodule. ⑤ Clear malignant ultrasound characteristics of the thyroid nodule.Exclusion criteria: ① No preoperative ultrasound imaging examination of the thyroid nodule and cervical lymph nodes, or unclear malignant ultrasound characteristics of the thyroid nodule. ② Unknown preoperative fine needle aspiration cytology (FNAC) of the thyroid nodule, or unclear postoperative pathological diagnosis of the thyroid nodule. ③ Postoperative maximum diameter of the thyroid nodule >10 mm. ④ Unclear diagnosis of thyroid-related diseases.This study was approved by the Medical Ethics Committee of Sichuan Provincial People's Hospital with approval number Lunshen (Research) 2023 No.427.

### Analysis indicators and research methods

**Figure 1:**
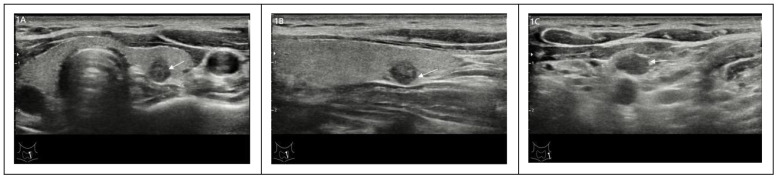
**(A-C)** A 30-year-old male with a thyroid tumor invading the capsule with ipsilateral cervical lymph node abnormalities was identified as a high-risk PTMC patient.

**Figure 2:**
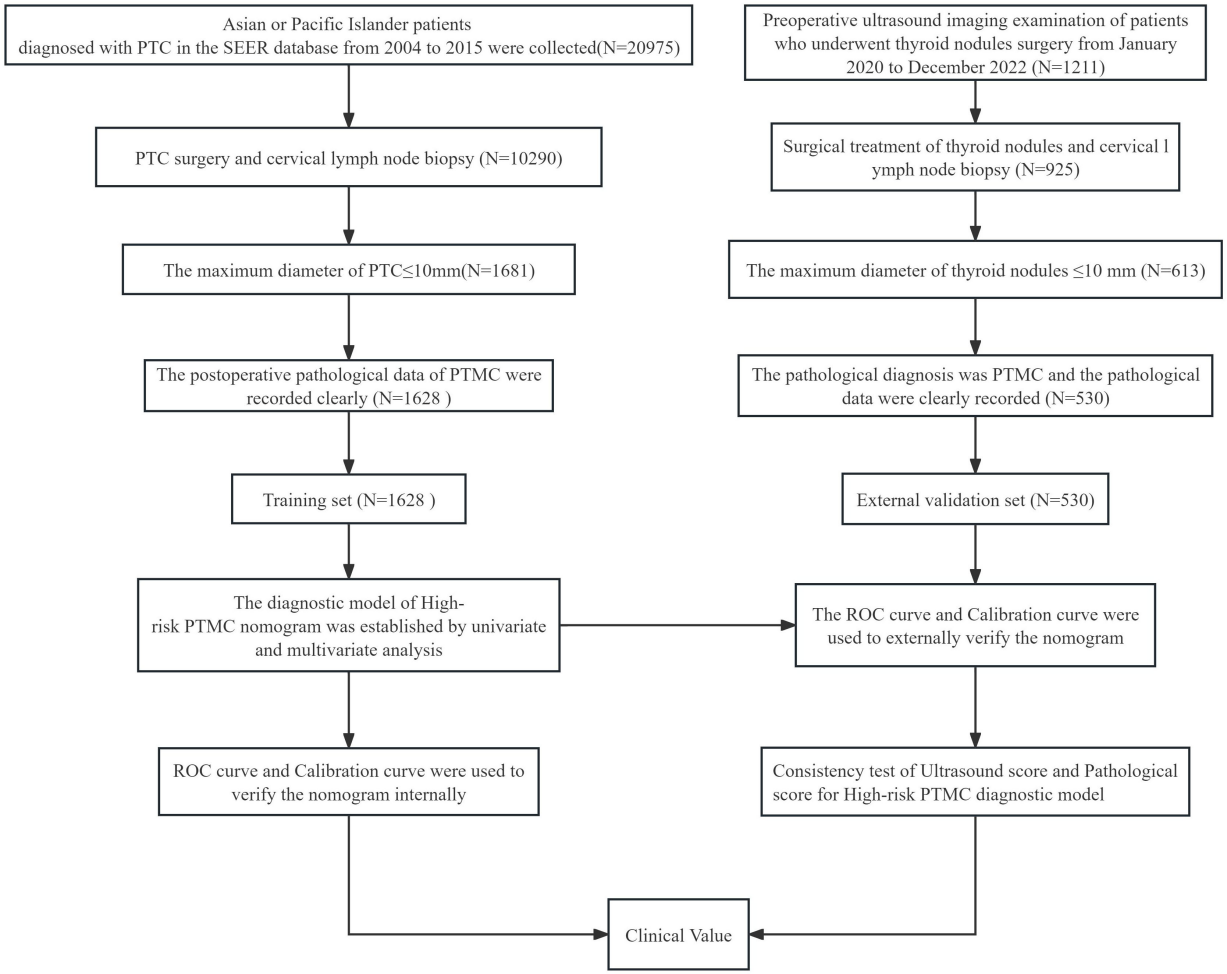
Research data screening process and research flow chart.

Clinical and pathological features, including sex, age, tumor maximum diameter, number of tumors, tumor invasive, and cervical lymph nodes, were included in this study. Pathological high-risk PTMC diagnosis standards (2–6).met any of the following criteria: ① PTMC pathology types were invasive subtypes. ② PTMC tumors significantly broke through the capsule and invaded the extraglandular area. ③ Pathological confirmation of cervical lymph node metastasis. In addition, Pathological high-risk PTMC diagnostic criteria was used as the Gold Standard for the diagnosis of this study.Ultrasonic imaging features: the number of lesions (two or more were multilocus lesions) and the maximum diameter (mm) of target tumor lesions were recorded. According to the relationship between the tumor and thyroid capsule, we determined whether the tumor had suspicious capsule invasion and extraglandular invasion and whether the cervical lymph nodes were abnormal according to the characteristics of the cervical lymph nodes. Typical images are shown in **Figures 1A–C**, and all ultrasonic images were judged by two older ultrasound physicians. A nomogram model of high-risk PTMC diagnosis was established based on clinical pathological characteristics, and then, the ultrasonic high-risk PTMC was indirectly evaluated according to the clinical pathological characteristics of thyroid and cervical lymph node ultrasound examination of imaging characteristics. Furthermore, the feasibility and reliability of preoperative ultrasound imaging features in diagnosing high-risk PTMC were verified through comparison with pathological high-risk PTMC.The specific data screening and research process are shown in **Figure 2**.

### Statistical methods

All test data in this study depended on SPSS 26.0 and R Studio software (version number: 2023.03.0 + 386) for statistical analysis and drawing. *P*<0.05 (bilateral inspection) represents statistical significance. Counting data were represented by the number of cases (n) or percentage (%), and intergroup comparisons were made using χ 2 Inspection. Normally distributed econometric data are represented by the mean ± standard deviation (x ± s), and independent sample ***t*** tests were used to compare differences between groups. SPSS 26.0 software was used for single factor regression analysis to screen statistically significant predictive factors for intergroup differences. Then, we incorporated them into multivariate logistic regression analysis, and a logistic regression model was established. The predictive factors in the multivariate logistic regression model were calculated to generate a nomogram using the R studio software package "rms" (version 6.2-0). The model was resampled 1000 times using the bootstrap method for internal validation, and a calibration curve was drawn to evaluate the model's calibration. The R studio software package "pROC" (version 1.18.0) was used to draw a nomogram total score to predict the ROC curve of high-risk PTMC. Using SPSS 26.0 software, the pathological characteristics of cases were used as an external validation set to test the predictive ability of the nomogram score (pathological score) for high-risk PTMC. Additionally, the ultrasound imaging features of cases were used as an external validation set to test the predictive ability of the nomogram score (ultrasound score) for high-risk PTMC before surgery. Finally, patients were divided into a pathological high-risk group, pathological low-risk group, ultrasound high-risk group, and ultrasound low-risk group based on the optimal threshold for scoring to conduct Spearman rank correlation analysis and Kappa consistency test on the ultrasound interpretation scores and pathological interpretation results of the external validation set separately.

## Results

### Basic characteristics of the research subjects

In the SEER database, there were a total of 20,975 patients diagnosed with PTC in the Asian population from 2004 to 2015. A total of 1628 PTMC patients who met the criteria were included in this study, including 275 males and 1353 females, 846 patients aged >45 years old, and 782 patients aged ≤ 45 years old. A total of 530 PTMC patients who met the research conditions of Sichuan Provincial People's Hospital from 2020 to 2022 were selected, including 168 males, 362 females, 214 patients over 45 years old, and 316 patients ≤ 45 years old. The specific clinical information is shown in **Table 1**.

**Table 1:** Basic clinicopathological or ultrasonographic features of PTMC patients [case ( % )].

Clinicopathological Ultrasonographic Features	SEER Training Set (N=1628)		χ2/t	*P value*	External Validation Set (N=530)		χ2/t	*P value*
	High-Risk	Low-Risk			High-Risk	Low-Risk		
Sex			38.848	0.00			13.377	0.00
Female	602 (37.0)	751 (46.1)			171 (32.3)	191 (36.0)		
Male	179 (11.0)	96 (5.9)			108 (20.4)	60 (11.3)		
Age			146.39	0.00			9.797	0.00
≤45year	497 (30.5)	285 (17.5)			184 (34.7)	132 (24.9)		
≤45year	284 (17.4)	562 (34.5)			95 (17.9)	119 (22.5)		
Tumor Maximum Diameter	7.62 ± 2.30mm	5.31 ± 2.88mm	17.942	0.00	7.45 ± 1.68mm	6.90 ± 1.81mm	3.648	0.00
Tumor Number			0.06	0.81			2.536	0.11
Single focus	519 (31.9)	558 (34.3)			180 (34.0)	145 (27.4)		
Multiple focus	262 (16.1)	289 (17.8)			99 (18.7)	106 (20.0)		
Tumor Invasive			166.939	0.00			104.807	0.00
Limited within the thyroid gland	527 (32.4)	786 (48.3)			172 (32.5)	246 (46.4)		
Invasion of the capsule or Extraglandular invasion	254 (15.6)	61 (3.7)			107 (20.2)	5 (0.9)		
Cervical Lymph Node			642.468	0.00			368.879	0.00
Normal	303 (18.6)	821 (50.4)			35 (6.6)	241 (45.5)		
Abnormal	478 (29.4)	26 (1.6)			244 (46.0)	10 (1.9)		

### Univariate and multivariate analysis of the training set

The results of univariate analysis showed that the indicators with statistically significant differences were sex, age, tumor maximum diameter, tumor invasive and cervical lymph nodes, as shown in **Table 1**. Incorporating these indicators into multivariate logistic regression analysis, the results revealed that sex, age, tumor maximum diameter, tumor invasive, and cervical lymph nodes were all independent predictors of high-risk PTMC occurrence (*P*<0.05). Among them, young males, abnormal cervical lymph nodes, and obvious tumor invasion were the most important risk factors for the occurrence of high-risk PTMC (**Table 2**).

**Table 2:** Polytomous odds ratios of high-risk papillary thyroid microcarcinoma diagnostic model.

Variables	Odds ratio (95% CI)	*P* value
	Category Optimization	
Sex (Male vs Female)	3.987(2.589 to 6.140)	< 0.01
Age		< 0.01
≤45year	8.690(6.143 to 12.292)	
≤45year	1	
Tumor Maximum Diameter(mm)	1.480(1.387 to 1.580)	< 0.01
Tumor Invasive		< 0.01
Limited within the thyroid gland	1	
Invasion of the capsule or extraglandular invasion	6.243(4.041 to 9.645)	
Cervical Lymph Node		< 0.01
Normal	1	
Abnormal	84.998(50.869 to 142.024)	
C statistic	0.947 (0.936 to 0.958)	< 0.01

### Construction and validation of a high-risk PTMC diagnostic model

**Figure 3:**
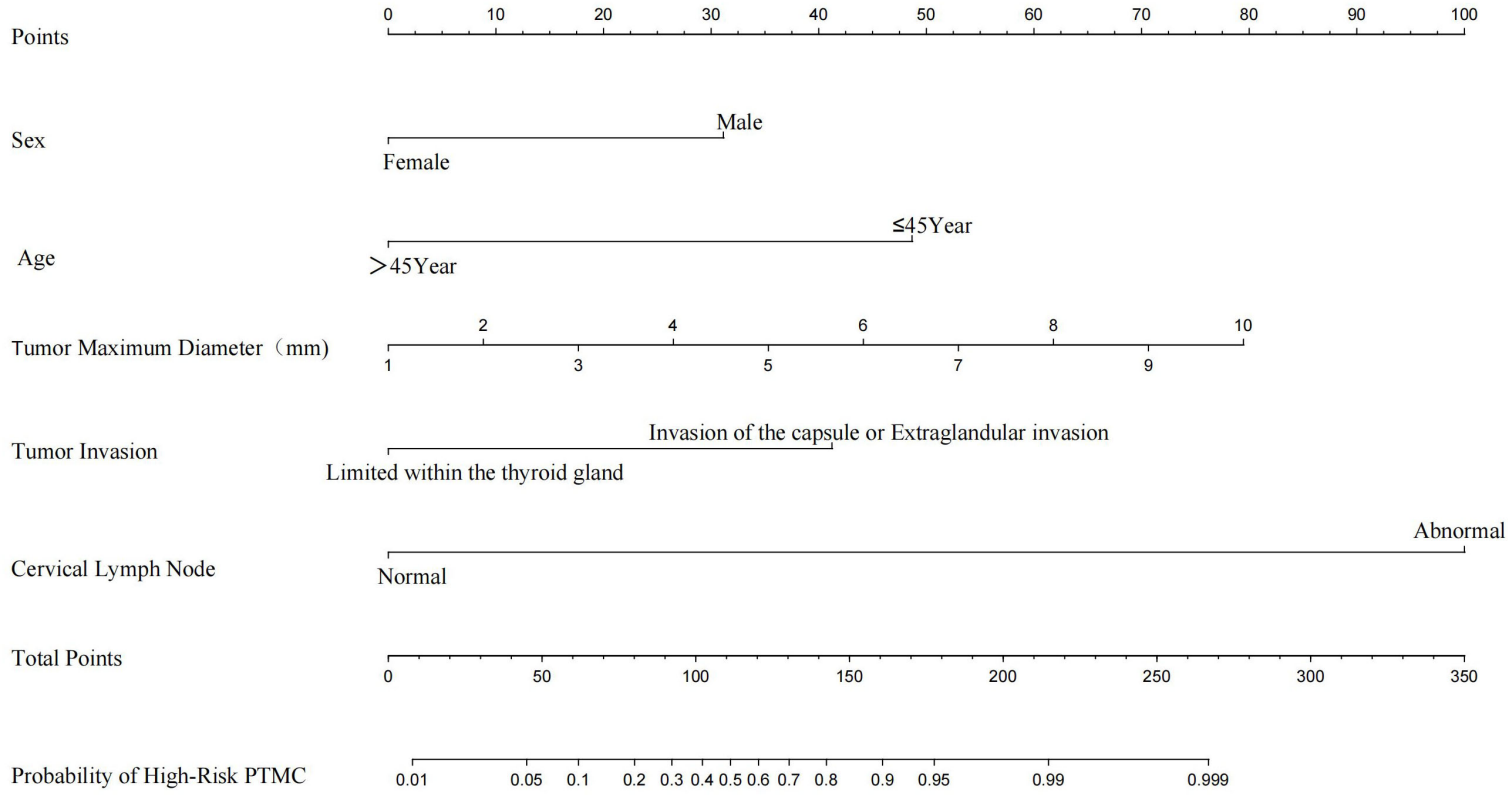
High-risk PTMC nomogram diagnostic model.

**Figure 4:**
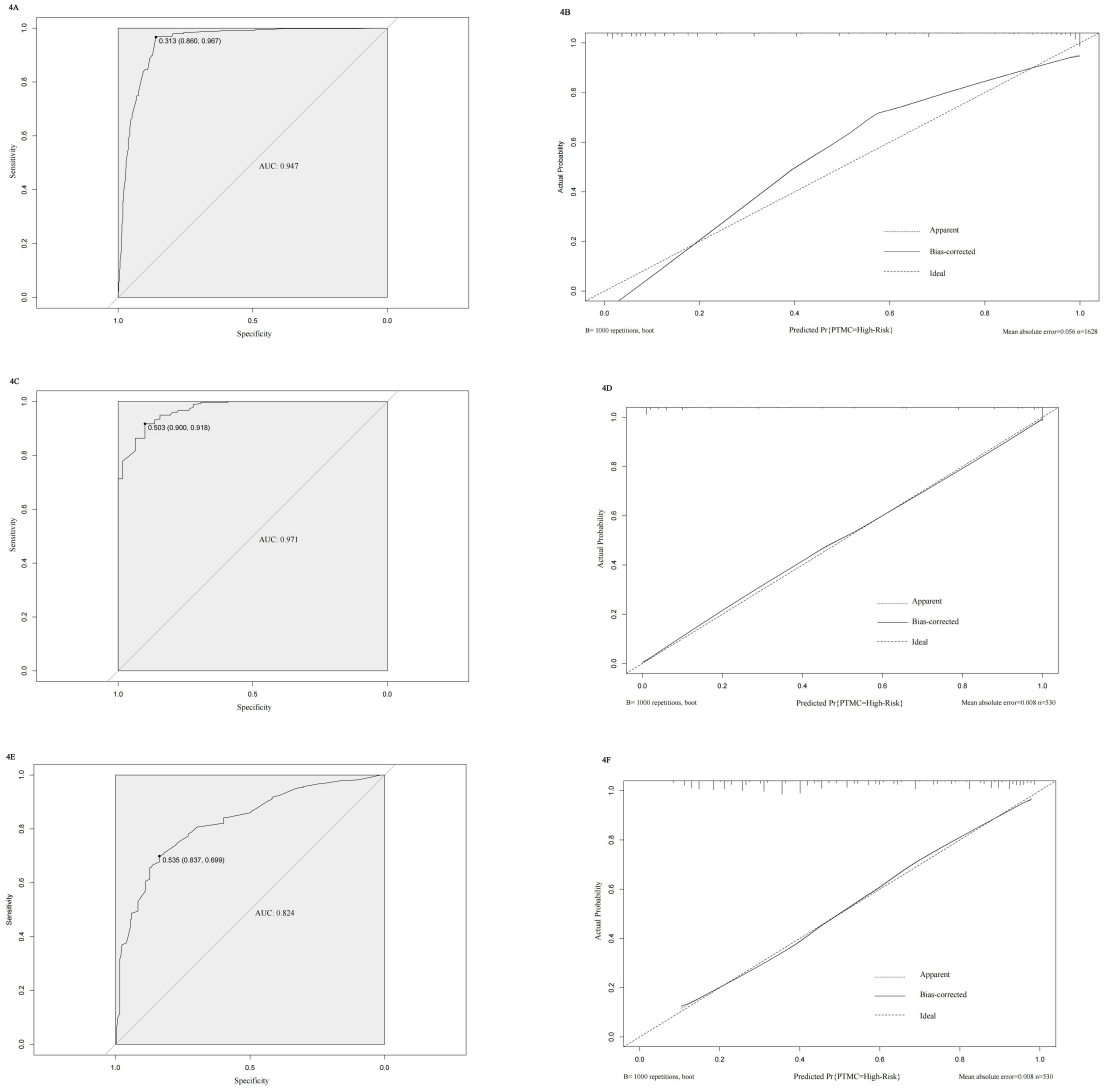
**(A-F)** Validation results of the model in internal and external validation sets. **(A)** ROC curve of internal training set. **(B)** Calibration curve of internal training set. **(C)** ROC curve of Pathological feature scores in external validation set. **(D)** Calibration curve of Pathological feature scores in external validation set. **(E)** ROC curve of Ultrasound image feature scores in external validation set. **(F)** Calibration curve of Ultrasound image feature scores in external validation set.

We constructed a nomogram diagnostic model for high-risk PTMC based on the predictive factors selected through multivariate logistic regression analysis in the training set (**Figure 3**). The C-index of this nomogram was 0.947, and the area under the ROC curve (AUC) was 0.947 (**Figure 4A**). The nomogram score under the optimal cutoff value was 92 points, with a sensitivity of 96.7% and a specificity of 86.0%. The model was internally validated using 1000 bootstrap resampling methods, and the calibration curve showed that the predicted results of this nomogram had a high degree of consistency with the actual situation (**Figure 4B**). In the external validation set, we calculated the risk score for each patient based on their pathological characteristics and ultrasound imaging characteristics and drew a calibration curve. The calibration curve showed that the predicted high-risk PTMC based on pathological feature scores had high consistency with the actual situation (**Figure 4D**), and the ROC curve indicated an AUC value of 0.971 [95% CI (0.960, 0.982), **Figure 4C**]. The consistency between the predicted results of high-risk PTMC based on ultrasound image feature scores and the actual situation was also ideal (**Figure 4F**), and the ROC curve results showed an AUC value of 0.824 [95% CI (0.789, 0.860), **Figure 4E**].

### Clinical application value of the high-risk PTMC diagnostic model

Based on the nomogram, the scores for each variable are as follows: Sex (male 31 points, female 0 points), Age (≤45 years 48 points, >45 years 0 points), Tumor maximum diameter (0 to 79 points), Tumor Invasive (limited within the thyroid gland 0 points, invasion of the capsule or Extraglandular invasion 41 points), Cervical Lymph Nodes (normal 0 points, abnormal 99 points).The sum of the scores of each variable represents the total score for Pathological Features or Ultrasound Features.According to the optimal cutoff value (92 points) scored on the nomogram, patients were divided into a Pathological high-risk group (Based on a total pathological feature score>92 points), Pathological low-risk group (Based on a total pathological feature score ≤ 92 points), Ultrasound high-risk group (Based on a total ultrasound feature score>92 points), and Ultrasound low-risk group (Based on a total ultrasound feature score≤ 92 points). According to the Kappa consistency test results, the κ value was 0.758 (*P*<0.001, **Table 3**), revealing high consistency between the two scoring methods.

**Table 3:** Consistency test based on pathological feature score and ultrasound image feature score [case ( % )].

Ultrasound Score	Pathological Score			*κ*value	*P* value
	Low-Risk	High-Risk	Total		
Low-Risk	145 (27.4)	54 (10.2)	199 (37.5)	0.758	<0.01
High-Risk	3 (0.6)	328 (61.9)	331 (62.5)		
Total	148 (27.9)	382 (72.1)	530 (100)		

**Figure 5:**
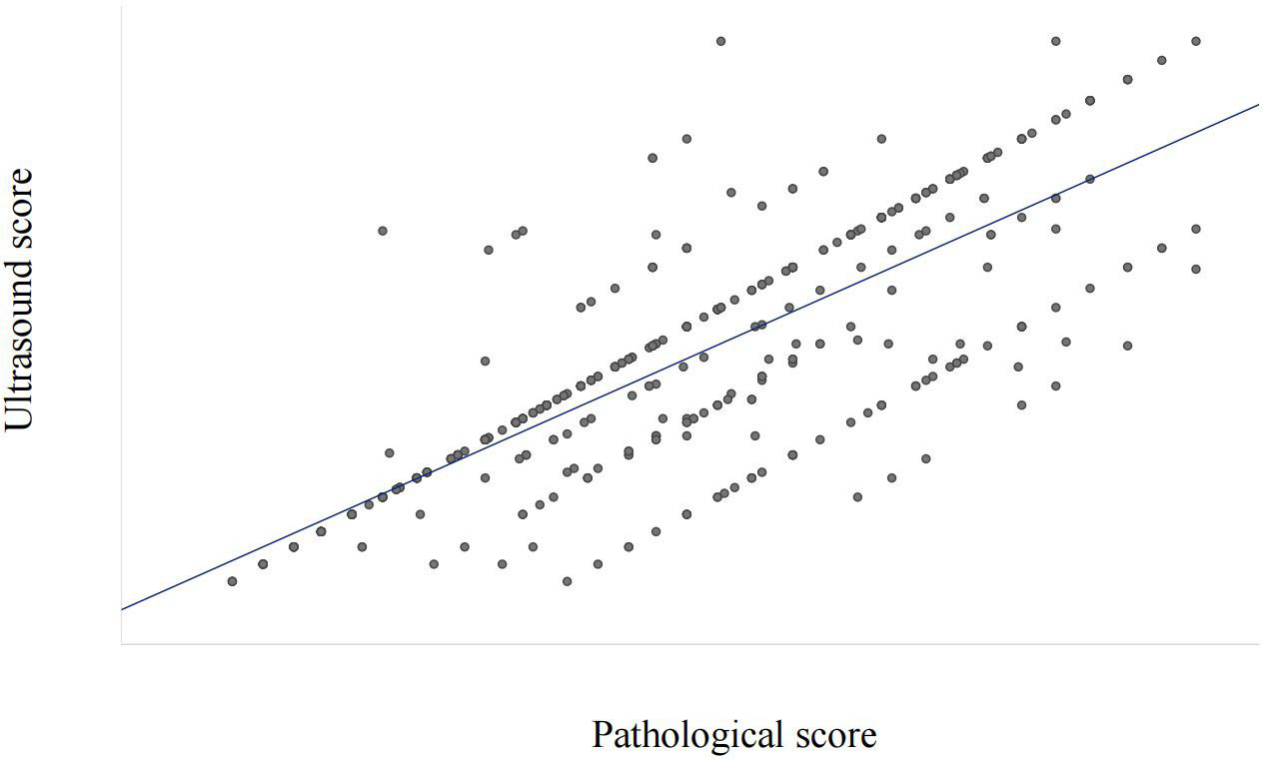
Correlation analysis based on pathological score and ultrasound score.

To clarify the reliability and clinical practicality of scoring based on ultrasound image features, the consistency between pathological feature scoring and ultrasound image feature scoring was tested. The Spearman rank correlation analysis results showed a high correlation between pathological feature scores and ultrasound image feature scores, with a correlation coefficient of 0.777 (*P*<0.001, **Figure 5**).

These results further indicate that preoperative ultrasound imaging features have clinical feasibility and reliability for the diagnosis of high-risk PTMC and provide a diagnostic basis for selecting optimal treatment strategies for PTMC.

## Discussion

Although the incidence of thyroid cancer in various countries has increased in recent years, the overall mortality rate has tended to be stable. PTC accounts for more than 80% of thyroid cancer cases. Among the new cases, the fastest growing type is PTMC, accounting for approximately half of the increment of thyroid cancer (4, 7). PTMC is usually considered a low-risk tumor, but the invasive subtype of PTMC does have a risk of local recurrence and distant metastasis (8, 9). At present, the thyroid imaging reporting and data system (TI-RADS) is widely used for the preoperative assessment of thyroid tumor malignancy risk (4, 10). However, it cannot further distinguish high-risk and low-risk PTMC, resulting in a large number of low-risk PTMC patients with higher TI-RADS classification undergoing invasive surgical treatment. Therefore, it is necessary to evaluate high-risk and low-risk PTMC patients to develop personalized treatment plans.

Previously, the vast majority of research diagnostic modelsfocused on predicting the risk of benign and malignant thyroid nodules, cervical lymph node metastasis and postoperative recurrence (11, 12). However, there are few preoperative evaluations of the clinical high risk of PTMC. Yan Z predicted the invasive of PTMC by combining ultrasound and the WNT10A gene (13). Their research showed that risk factors for PTMC invasive include age<45 years old, maximum nodule diameter>7 mm, microcalcification, and high expression of US-LNM and WNT10A. These results were basically consistent with the conclusions of our study. Moreover, Zhang Y suggested that the combination of serum microRNAs and ultrasound could serve as a predictive indicator for the diagnosis and prognosis of PTMC (14). However, these studies did not distinguish between high-risk and low-risk PTMC. Other PTMC diagnostic models constructed through metabolomics methods based on NMR and serum N-glycosides have failed to diagnose high-risk PTMC (15, 16). The C-index of the high-risk PTMC diagnostic model established in our study reached 0.947, indicating that the model had excellent clinical discrimination and predictive ability. However, it is worth noting that the screening analysis and model construction of these high-risk predictive factors were based on postoperative pathological characteristics, which to some extent limited the reference value of this diagnostic model for preoperative planning and development of personalized treatment strategies.

Among the indicators included in the model constructed in this study, the tumor maximum diameter, extraglandular invasion, and abnormal cervical lymph nodes could be indirectly judged through ultrasound imaging, which would then predict whether PTMC patients were at high risk before surgery. Furthermore, the results of our study indicated that there was high consistency between scoring based on ultrasound and scoring based on pathological features. The value of κ reached 0.758 and had good discrimination, which confirmed the preoperative application value of this model. Shen K suggested that a preoperative and postoperative recurrence risk model for PTC could be constructed based on clinical features such as sex and age, as well as ultrasound features such as extraglandular invasion and cervical lymph node abnormalities (17). Their model was consistent with the predictive factors selected in our study, but its AUC value was only 0.777, which was lower than that in our study. Although some studies have suggested that multifocal nodules, location, and extraglandular expansion are also high-risk factors affecting PTMC (18–20), we did not adopt them in our study, which may be due to our biased selection of research data, resulting in statistical results not being included. Moreover, Liu FF found through a study of 719 non-high-risk PTMC patients undergoing first-time surgery that the cervical lymph node metastasis rate in the 30-39-year-old group was higher than that in the 50-59-year-old group (21). The lymph node metastasis rate of PTMC with capsule invasion was significantly higher than that of PTMC without capsule invasion, indicating the correctness of including these high-risk indicators in the diagnosis of high-risk PTMC. At the same time, there are differences in the sensitivity, specificity, and accuracy of ultrasound imaging in evaluating extraglandular invasion and cervical lymph node abnormalities (22–24), but many studies have revealed that using ultrasound imaging features to indirectly evaluate pathological features has clinical feasibility and consistency (25, 26). Therefore, predicting high-risk PTMC through preoperative ultrasound image features had certain clinical feasibility, but there were subjective differences in the interpretation of ultrasound image features among different physicians, which affected the final diagnostic performance of the model.

The advantage of this study was that the SEER database included a large number of clinical and pathological data of PTMC patients, which was more reliable than previous small-scale retrospective results. Therefore, we could better study high-risk PTMC with larger data. Meanwhile, there were still the following limitations in our study: ① This study was based on the SEER public database for data analysis and model construction, and some potential risk factors were not recorded in the SEER database, including thyroid function status, tumor invasion and expansion status, neck radiation history, etc. ② The sample size included in the external validation set in this study was relatively small, and there was selection bias. Therefore, it is still necessary to verify the effectiveness of the model with larger sample size data in the future.

## Conclusions

In conclusion, this diagnostic model for indirectly evaluating high-risk PTMC that we constructed in our study based on preoperative ultrasound imaging features has potential clinical application value and good predictive effects and provides diagnostic references for personalized treatment strategies in clinical practice.

## Data Availability

The original contributions presented in the study are included in the article/**Supplementary Material**. Further inquiries can be directed to the corresponding author.
